# Non-invasive Follicular Thyroid Neoplasm With Papillary-Like Nuclear Features (NIFTP) Arising in Struma Ovarii: A Pathologist’s Perspective

**DOI:** 10.7759/cureus.92857

**Published:** 2025-09-21

**Authors:** Hasnae Ismaili, Sanae El Bardai, Majda Bendahhou Idrissi, Chahrazed Bouchikhi, Nadia Alaoui Ismaili, Nawal Hammas, Laila Chbani, Layla Tahiri Elousrouti

**Affiliations:** 1 Pathology, University Hospital Hassan II, Fez, MAR; 2 Obstetrics and Gynecology, University Hospital Hassan II, Fez, MAR; 3 Nuclear Medicine, University Hospital Hassan II, Fez, MAR

**Keywords:** braf, niftp, ovary, struma ovarii, teratoma

## Abstract

Struma ovarii is a rare monodermal ovarian teratoma predominantly composed of thyroid tissue. It is usually benign, although malignant transformation is occasionally observed. Non-invasive follicular thyroid neoplasm with papillary-like nuclear features (NIFTP) is a recently defined non-malignant entity with indolent behavior, and its emergence in struma ovarii is very uncommon. It requires careful histological analysis, and because of the unusual occurrence of this tumor, its management and follow-up remain challenging and debated. We report a case of NIFTP arising in struma ovarii in a 48-year-old patient. To the best of our knowledge, this is the second case report of this nature.

## Introduction

Struma ovarii is an uncommon monodermal ovarian teratoma, primarily composed of thyroid tissue (>50%), and accounts for approximately 2-3% of all ovarian teratomas [1]. While it is generally benign, cases of malignant transformation have been reported [2]. Non-invasive follicular thyroid neoplasm with papillary-like nuclear features (NIFTP) is a newly recognized non-malignant entity characterized by indolent behavior [3], and its emergence in struma ovarii is very uncommon.

Clinically, patients often present with nonspecific symptoms, and while imaging studies assist in characterizing the ovarian mass, definitive diagnosis relies on pathological examination [4]. The diagnosis of NIFTP specifically requires histological analysis of the entire excised tumor using well-defined inclusion and exclusion criteria.

Surgical resection of the ovarian mass remains the mainstay of treatment; however, due to the rarity of this presentation, there is no consensus on the optimal surgical approach, the extent of surgery, or the appropriate postoperative follow-up. This lack of standardized guidelines underscores the need for further case reporting and collaborative studies to refine the management and follow-up strategies for NIFTP arising in struma ovarii.

We present a case of NIFTP arising in struma ovarii, which was unexpectedly discovered during histopathologic examination. To the best of our knowledge, this is the second case report of this nature.

## Case presentation

A 48-year-old woman presented to the gynecology department with a three-month history of pelvic heaviness. Her medical history was unremarkable, and there was no family history of gynecological malignancies. On abdominal examination, a palpable mass with hypogastric tenderness was noted. Ultrasound and CT imaging revealed a large solid-cystic mass measuring 10.89 × 10.02 cm in the right adnexal region. The patient subsequently underwent a unilateral salpingo-oophorectomy with omentectomy.

**Figure 1:**
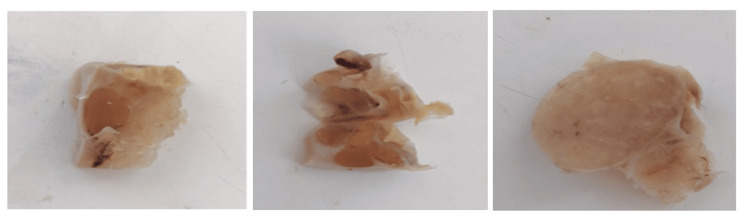
Gross examination of the ovary revealing a multiloculated complex cystic lesion that grossly resembled thyroid tissue.

Gross examination of the resected ovary showed a multiloculated complex cystic lesion containing solid areas, with a smooth and intact capsule. On the cut section, the mass grossly resembled thyroid tissue (Figure 1).

**Figure 2:**
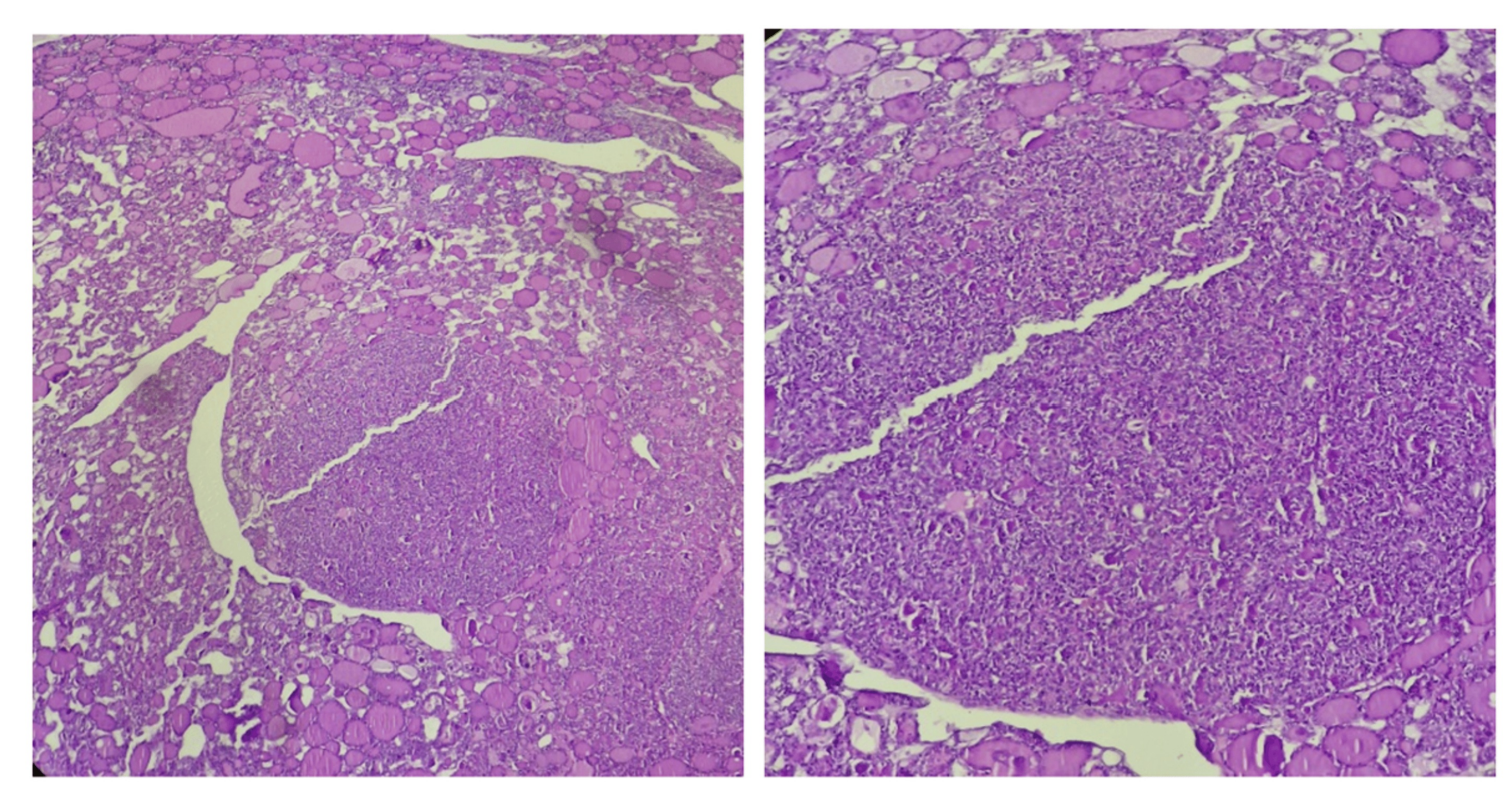
Histopathological examination revealed benign thyroid follicles with abundant colloid with a single focus characterized by the formation of crowded abnormal thyroid follicular cells.

**Figure 3:**
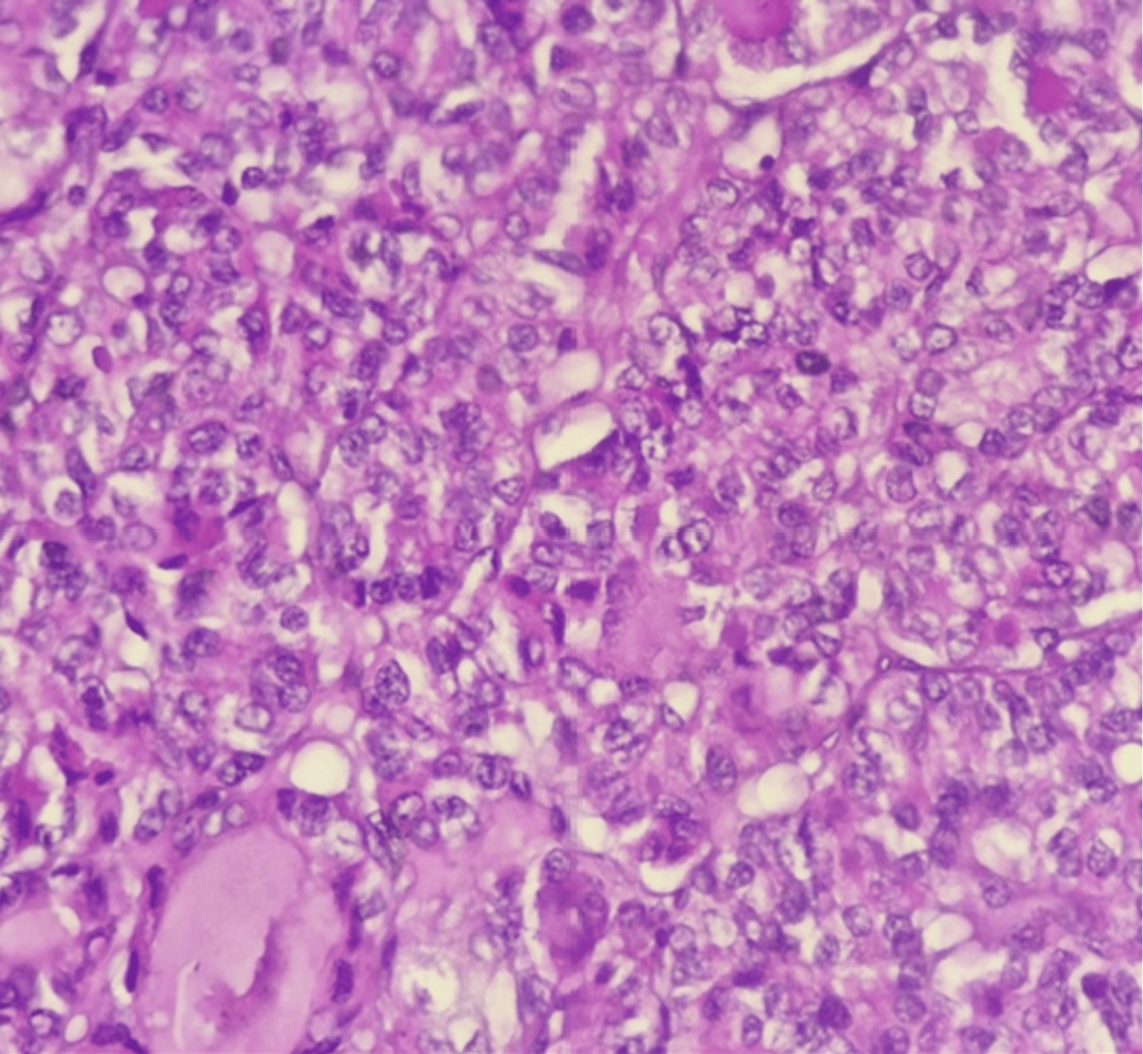
The cells exhibited enlarged, irregular nuclei with pale (optically clear) chromatin and the presence of nuclear grooves.

Histopathological analysis demonstrated benign thyroid follicles with abundant colloid, alongside a single well-circumscribed focus of crowded, abnormal thyroid follicular cells (Figure 2). These cells were organized exclusively in a vesicular architectural pattern and displayed enlarged, irregular nuclei with pale (optically clear) chromatin and nuclear grooves (Figure 3). No capsular or vascular invasion, high-grade features such as mitotic activity or necrosis, or psammoma bodies were identified. A diagnosis of NIFTP within struma ovarii was made.

**Figure 4:**
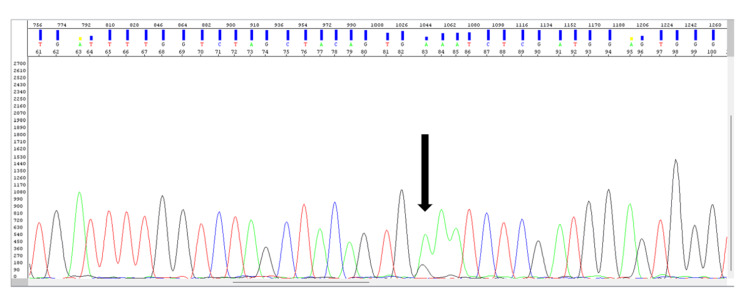
Molecular study revealing the presence of the C.1801 A>G p.K601E mutation in exon 15 of the BRAF gene (black arrow).

To support the diagnosis, a molecular study was conducted, revealing the presence of the c.1801A>G p.K601E mutation in exon 15 of the BRAF gene (Figure 4).

Because of the unusual occurrence of this tumor, a multidisciplinary meeting was held, leading to the decision to perform a total thyroidectomy followed by radioiodine therapy.

## Discussion

Struma ovarii is a rare monodermal ovarian teratoma that accounts for 0.5-1% of ovarian tumors and 3-5% of ovarian teratomas [5]. It is predominantly benign, with malignant transformation occurring in about 5% of cases [2]. First described by Bottlin in 1888 and later studied by Ludwig Pick, struma ovarii is characterized by the overgrowth of thyroid tissue within the ovarian teratoma [6].

The tumor typically affects women in their fifth and sixth decades of life [7]. The diagnosis of struma ovarii is often incidental, occurring during imaging or surgical exploration for a pelvic mass [8]. Symptoms, when present, may include abdominal pain, a detectable mass, ascites, or vaginal bleeding [9]. Clinical hyperthyroidism is observed in 5-8% of patients [10]. Histopathological examination shows thyroid follicles containing colloid. Immunohistochemical staining may be performed to confirm the thyroid tissue origin, showing positivity for thyroglobulin and TTF-1 [11].

NIFTP is now considered a non-malignant indolent lesion [3]. Accurate diagnosis requires rigorous histological evaluation, including encapsulation, a purely follicular architecture, and papillary thyroid carcinoma-type nuclear features, while ensuring the absence of capsular or vascular invasion, high-grade characteristics such as mitotic activity or necrosis, and any significant solid growth component.

The majority of NIFTPs are driven by RAS mutations or codon 601 BRAF mutations, while the presence of the BRAF p.V600E mutation serves as an exclusion criterion for NIFTP according to the new WHO classification of thyroid neoplasms. However, molecular testing is not considered essential for diagnosing NIFTP, yet the detection of a BRAF V600E mutation automatically excludes the diagnosis. This exclusion criterion is upheld in the 2022 WHO classification and could encourage systematic testing for BRAF mutations (using immunohistochemical or molecular techniques) before confirming a diagnosis of NIFTP [12].

It is important to remember that this is an exclusion criterion, and the absence of a BRAF V600 mutation is not sufficient to provide reassurance in the presence of a challenging diagnosis of an encapsulated follicular tumor. Rigorous application of all diagnostic criteria for NIFTP, particularly the absence of invasive features or any suspicious indications of invasion, remains paramount [12].

Complete tumor resection is considered the treatment of choice, with postoperative strategies varying based on the case. Although there are no established guidelines for preventive thyroidectomy, some authors advocate performing thyroidectomy following the diagnosis of benign struma ovarii, whereas in cases of malignant struma ovarii, they recommend iodine radioablation therapy. Some studies highlight the benefit of thyroidectomy and radioactive iodine, reporting recurrence-free outcomes even decades later [13].

It is advisable to assess thyroid function tests, including thyroglobulin levels, in patients with suspected ovarian tumors before surgery. Similarly, in individuals with thyroid disorders, evaluating the pelvic organs, particularly the ovaries, is recommended.

Long-term follow-up is recommended to monitor prognosis and detect potential recurrences. While NIFTP demonstrates excellent prognosis in thyroid gland cases, its behavior in struma ovarii remains uncertain, warranting further investigation [14].

Pathologists should be aware of this entity and exclude it in the workup of ovarian teratomas. Multidisciplinary management involving endocrinologists, gynecologists, and oncologists is crucial for optimal care.

## Conclusions

NIFTP is a recently recognized thyroid tumor entity characterized by an indolent clinical course and generally associated with a favorable prognosis. Exceptionally, NIFTP can arise within struma ovarii, a rare monodermal ovarian teratoma predominantly composed of thyroid tissue. Given the rarity of this occurrence, the diagnosis requires a high index of suspicion and is established through strictly defined inclusion and exclusion criteria.

From a molecular perspective, NIFTP is most frequently associated with RAS mutations, although other genetic alterations, such as the BRAF K601E mutation, may rarely be identified.

There is currently no established standard treatment protocol for NIFTP arising within struma ovarii. The rarity of this condition, coupled with the limited number of reported cases, poses challenges in determining the optimal surgical approach and postoperative management strategy. This highlights the need for multicenter studies and further research to gather robust evidence and develop consensus guidelines that will aid clinicians in the effective management and follow-up of patients with this rare entity.
